# The Pharmacological Evidences for the Involvement of AhR and GPR35 Receptors in Kynurenic Acid-Mediated Cytokine and Chemokine Secretion by THP-1-Derived Macrophages

**DOI:** 10.3390/molecules30153133

**Published:** 2025-07-26

**Authors:** Katarzyna Sawa-Wejksza, Jolanta Parada-Turska, Waldemar Turski

**Affiliations:** 1 Department of Virology and Immunology, Maria Curie-Sklodowska University, 20-033 Lublin, Poland; katarzyna.sawa-wejksza@mail.umcs.pl; 2 Department of Rheumatology and Connective Tissue Diseases, Medical University of Lublin, 20-090 Lublin, Poland; jolanta.parada-turska@umlub.pl; 3 Department of Neurotoxicology, Institute of Agricultural Medicine, 20-090 Lublin, Poland

**Keywords:** kynurenic acid, THP-1, macrophages, aryl hydrocarbon receptor, G protein-coupled receptor 35, cytokines, immunomodulation

## Abstract

Kynurenic acid (KYNA), a tryptophan metabolite, possesses immunomodulatory properties, although the molecular mechanism of this action has not yet been resolved. In the present study, the effects of KYNA on the secretion of selected cytokines and chemokines by macrophages derived from the human THP-1 cell line are investigated. Furthermore, the involvement of the aryl hydrocarbon receptor (AhR) and the G protein-coupled receptor 35 (GPR35) in mediating the effects of KYNA was examined. In lipopolysaccharide (LPS)-stimulated THP-1-derived macrophages, KYNA significantly reduced IL-6 and CCL-2, but increased IL-10 and M-CSF levels. AhR antagonist CH-223191 reduced the KYNA influence on IL-6, CCL-2, and M-CSF production, while the GPR35 antagonist, ML-145, blocked KYNA-induced IL-10 production. Furthermore, it was shown that THP-1 derived macrophages were capable of synthesizing and releasing KYNA and that its production was increased in the presence of LPS. These findings suggest that THP-1-derived macrophages are a source of KYNA and that KYNA modulates inflammatory responses predominantly through AhR and GPR35 receptors. Our study provides further evidence for the involvement of macrophages in immunomodulatory processes that are dependent on AhR and GPR35 receptors, as well as the potential role of KYNA in these phenomena.

## 1. Introduction

The metabolites produced from tryptophan via the kynurenine pathway are well known for significantly influencing various immune cells and contributing to numerous physiological and pathological conditions involving the immune system [1]. They are implicated in maintaining pregnancy by modulating maternal and fetal immune tolerance [2], regulate inflammatory responses [3], and support tissue homeostasis [4]. Additionally, they are related to tumorigenesis [5]. However, despite their involvement in such critical physiological processes, our understanding of the effects of individual kynurenine metabolites on specific immune cell types remains limited.

Kynurenic acid (KYNA) is a tryptophan metabolite formed via the kynurenine pathway. KYNA is produced endogenously from its immediate precursor kynurenine (L-KYN) by kynurenine aminotransferases. These enzymes are localized intracellularly, whereas the synthesized KYNA is liberated into the extracellular space and subsequently enters the bloodstream. KYNA is not accumulated in the body; instead, it is excreted mainly in urine in an unmetabolized form [6,7].

KYNA is known as an antagonist of ionotropic glutamate receptors and the alpha-7 nicotinic acetylcholine receptor, which are located mainly in the central nervous system. Since KYNA does not penetrate the blood–brain barrier sufficiently [8] and has been evidenced to act as an agonist of the aryl hydrocarbon receptor (AhR) and the G protein-coupled receptor 35 (GPR35), both of which are present in many peripheral cell types, also in immune cells [9], its influence on the physiology of cells located outside the brain has attracted increasing interest in recent years.

Importantly, both AhR and GPR35 are functional on macrophages. Macrophages are cells widely distributed throughout various tissues and body cavities, where they maintain homeostasis and regulate immune responses. They participate in key stages of inflammation, including pathogen recognition and the modulation of inflammatory processes through the secretion of various cytokines and chemokines [10]. Depending on environmental signals, macrophages are pro-inflammatory and specialize in host defense and tumor cell elimination, or are involved in tissue repair, wound healing, and the resolution of inflammation [11].

The relative ease of increasing the peripheral KYNA concentration and the broad range of molecular targets of KYNA raise important questions about its systemic effects. Its potential importance in digestive tract function, along with other health benefits involving metabolic disorders and cancer, has been extensively reviewed [12]. Although its anti-inflammatory effects, including protective effects in sepsis, have been repeatedly confirmed [13,14], the molecular mechanisms underlying its action on immune cells remain poorly understood. Therefore, in the present study, we evaluated the effect of KYNA on the secretion of selected cytokines and chemokines by activated macrophages derived from the THP-1 cell line in vitro. Moreover, as the macrophage response can be easily affected by environmental stimuli, we examined the influence of KYNA on resting or lipopolysaccharide (LPS)-stimulated THP-1-derived macrophages. Furthermore, the involvement of AhR and GPR35 in mediating the effects of KYNA was investigated. Our study aims to provide further evidence for the involvement of macrophages in immune processes that are dependent on AhR and GPR35. In addition, it intends to address the potential importance of endogenous and exogenous KYNA, which is an agonist for both receptors, in these phenomena.

## 2. Results

### 2.1. KYNA Production by THP-1-Derived Macrophages

**Figure 1:**
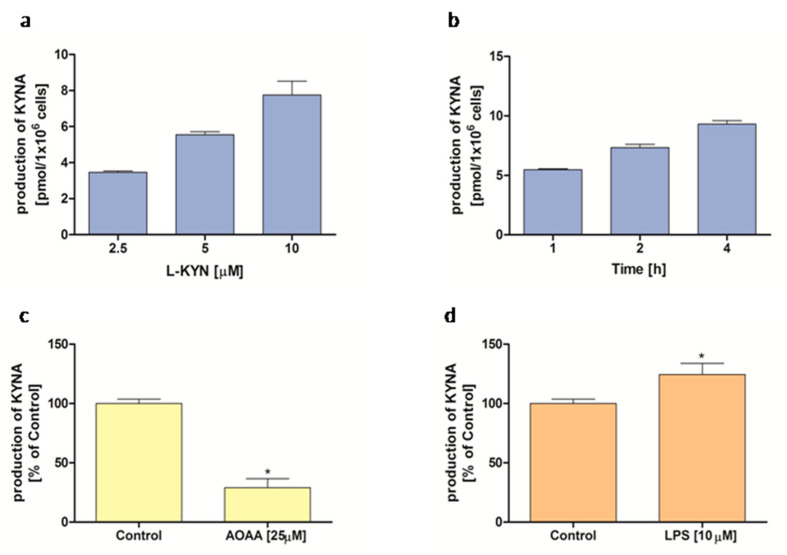
KYNA production by THP-1-derived macrophages: the influence of (**a**) concentration of substrate, L-KYN; (**b**) incubation time; (**c**) 25 µM AOAA; and (**d**) 10 μg/mL LPS on KYNA production in THP-1-derived macrophages. Data are presented as mean ± SD; (*) *p* < 0.05 vs Control.

The THP-1-derived macrophages incubated in the presence of L-KYN synthesized and liberated KYNA to the external milieu in a dose- and time-dependent manner (Figure 1a,b). The mean production of KYNA in the standard conditions (L-KYN 5 mM; 2 h incubation time) was 6.44 ± 1.17 pmol KYNA/5 × 10^5^ cells (*n* = 11). The KYNA production was significantly inhibited by aminooxyacetic acid (AOAA), a non-specific inhibitor of aminotransferases (Figure 1c). LPS at a concentration of 10 μg/mL enhanced the KYNA synthesis in the THP-1-derived macrophages (Figure 1d).

### 2.2. Influence of KYNA on the Production of Cytokines and Chemokines by THP-1-Derived Macrophages

To examine the influence of KYNA on cytokine secretion in physiological and inflammatory conditions, the THP-1-derived macrophages were incubated in standard and LPS-containing medium, respectively. It was found that, at the examined concentrations (50–250 μM), KYNA did not affect the production of any investigated cytokines in the standard conditions (medium without LPS) (Table S1, Supplementary Materials).

**Figure 2:**
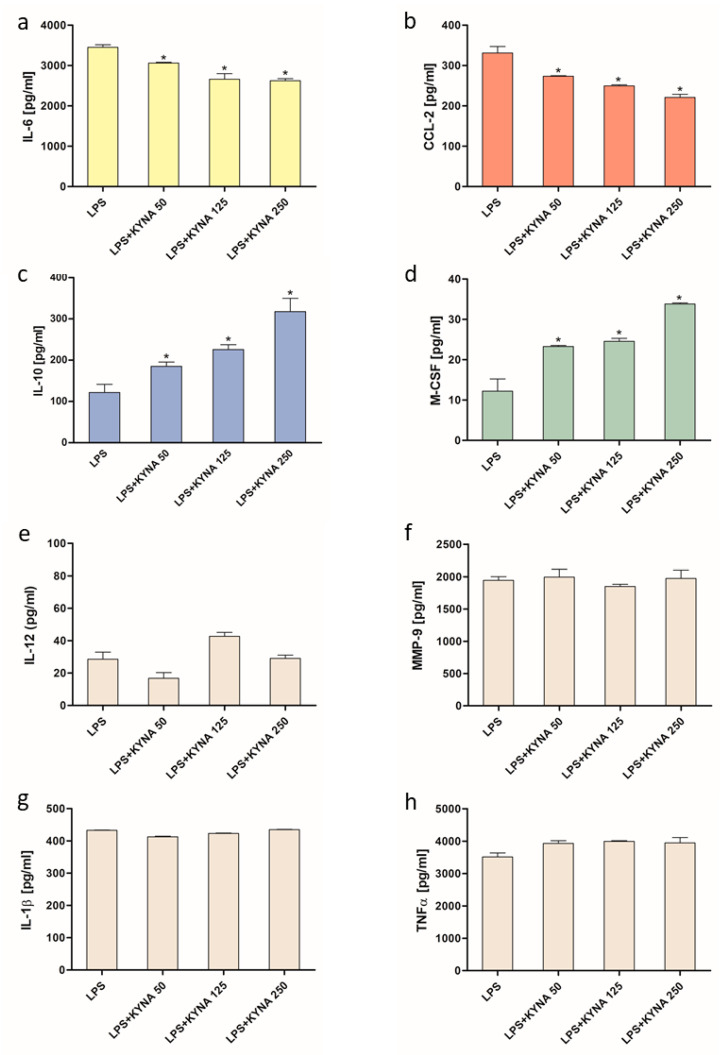
Influence of KYNA on the production of cytokines and chemokines (**a**) IL-6, (**b**) CCL-2, (**c**) IL-10, (**d**) M-CSF, (**e**) IL-12, (**f**) MMP-9, (**g**) IL-1β, and (**h**) TNFα, by THP-1-derived macrophages exposed to LPS. Data are presented as mean ± SD; (*) *p* < 0.05 vs. LPS.

However, in the THP-1-derived macrophages exposed to LPS, KYNA decreased the production of IL-6 and CCL-2 in a dose-dependent manner (Figure 2a,b) and enhanced the production of IL-10 and M-CSF dose-dependently (Figure 2c,d), but did not influence the LPS-induced production of IL-12, MMP-9, IL-1β, and TNFα (Figure 2e–h).

### 2.3. Involvement of GPR35 and AhR Receptors in the Action of KYNA on Cytokine Secretion

**Figure 3:**
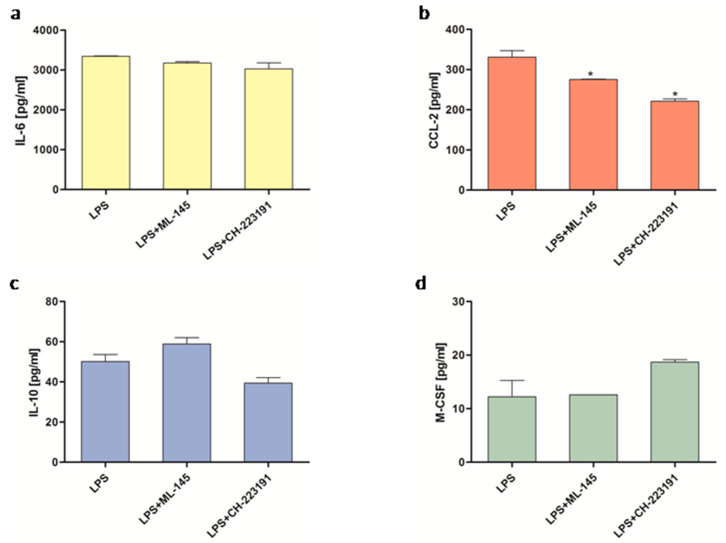
Influence of CH-223191 (an AHR antagonist) and ML-145 (a GPR35 antagonist) on the production of cytokines and chemokines (**a**) IL-6, (**b**) CCL-2, (**c**) IL-10, and (**d**) M-CSF by THP-1-derived macrophages. Data are presented as mean ± SD; (*) *p* < 0.05 vs LPS.

To investigate the mechanism by which KYNA influences the cytokine production, the THP-1-derived macrophages incubated with LPS were exposed to antagonists of GPR35 (ML-145) or AhR (CH-223191), either alone or in combination with KYNA. Four cytokines and chemokines were selected (IL-6, M-CSF, IL-10, CCL-2), as KYNA modulated their production in the THP-1-derived macrophages. It was found that both antagonists alone did not affect the release of the selected cytokines (Figure 3a–c), with the exception of CCL-2, the secretion of which was significantly reduced by both ML-145 and CH-223191 (Figure 3b).

**Figure 4:**
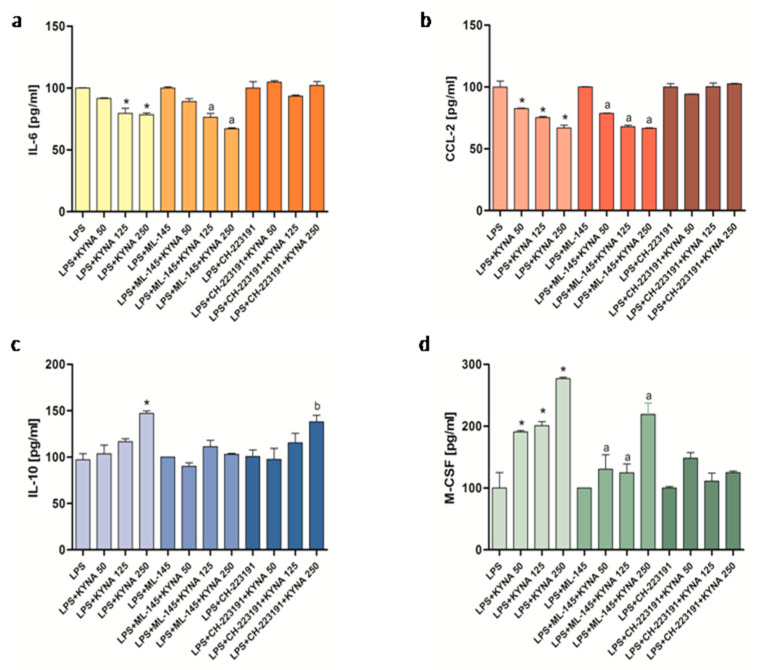
Influence of KYNA on the production of cytokines and chemokines (**a**) IL-6, (**b**) CCL-2, (**c**) IL-10, and (**d**) M-CSF by THP-1-derived macrophages in the presence of CH-223191 (an AHR antagonist) and ML-145 (a GPR35 antagonist). Data are presented as mean ± SD; (*) *p* < 0.05 vs. LPS group; (a) *p* < 0.05 vs. LPS + ML-145 group; (b) *p* < 0.05 vs. LPS + CH-223191 group.

CH-223191 (an AhR antagonist) significantly blocked the effect of KYNA exerted on IL-6 production, whereas ML-145 (a GPR35 antagonist) was ineffective in this respect (Figure 4a). A similar pattern was observed for CCL-2 (Figure 4b). In the case of IL-10, ML-145 significantly blocked the effect of KYNA exerted on its production, and CH-223191 was ineffective in this respect (Figure 4c). In turn, ML-145 partially reduced and CH-223191 completely inhibited the KYNA-induced enhancement of M-CSF release (Figure 4d).

## 3. Discussion

KYNA is known to influence a broad range of cellular targets. In the central nervous system, it antagonizes ionotropic glutamate receptors and the alpha-7 nicotinic acetylcholine receptor. In peripheral tissues, KYNA primarily acts as an agonist of the AhR and GPR35, both of which are expressed across multiple cell types [9]. Furthermore, KYNA has been postulated to interact with additional targets, including the hydroxycarboxylic acid receptor 3 and adrenoceptor alpha-2B [15], although there is no direct experimental evidence supporting this hypothesis.

The broad spectrum of KYNA targets raises important questions regarding its potential systemic effects, especially given that KYNA is constantly present in the human serum at concentration ranges from 0.030 to 0.050 μM [16] and is endogenously synthesized by several peripheral tissues, such as muscle, liver, kidney, pancreas, and endothelial cells [17].

Our study has also demonstrated that THP-1-derived macrophages are capable of producing KYNA from exogenously supplied L-KYN in a time and dose-dependent manner. The inhibition of this process by AOAA, a known aminotransferase inhibitor, confirms the enzymatic nature of KYNA synthesis. Moreover, we found that the LPS stimulation significantly enhanced the KYNA production in the THP-1-derived macrophages, which is consistent with other studies reporting elevated KYNA levels following immune activation. Increased KYNA concentration was reported in the plasma of LPS-treated pigs [18], in the medial pre-frontal cortex of mice treated with LPS [19], and in the plasma of mice subjected to chronic stress [20]. Similarly, in vitro activation of Toll-like receptors in human monocytes has been shown to upregulate KYNA production [21]. Despite the accumulating evidence linking inflammation with increased KYNA synthesis, the physiological and pathological relevance of this phenomenon needs further investigation.

Given that KYNA is present in various body compartments and its levels can be readily elevated, a key question arises regarding its effects on systemic pathophysiological processes, including immune regulation. KYNA has been consistently shown to exert systemic anti-inflammatory effects, providing protection in animal models of sepsis [14,22], and reducing inflammation in the gastrointestinal tract [23,24,25]. Although its anti-inflammatory effects, including protective role in sepsis, have been repeatedly confirmed [13,14], the molecular mechanisms underlying its effects on immune cells remain poorly understood. Therefore, in the present study, we evaluated the influence of KYNA on the secretion of selected cytokines and chemokines by THP-1-derived macrophages in vitro, a model widely accepted as representative of human macrophages [26]. As the KYNA impact on immune cells varies depending on the experimental conditions, we studied the effects of KYNA on THP-1-derived macrophages in both standard control and inflammatory conditions.

Our results showed that KYNA did not affect the secretion of any of the examined cytokines in the control conditions. On the other hand, upon LPS stimulation, KYNA significantly and dose-dependently decreased pro-inflammatory IL-6 and increased anti-inflammatory IL-10 cytokine secretion in the THP-1-derived macrophages. This finding aligns with previous studies showing that KYNA attenuates the inflammatory condition and possesses anti-inflammatory properties [20,27,28,29]. To further explore the immunomodulatory properties of KYNA, we investigated its impact on the production of CCL-2, a chemokine critical for the recruitment of monocytes and macrophages to sites of infection, which is crucial in initiation and sustaining inflammatory response. Elevated levels of CCL-2 are commonly observed in inflammatory diseases, such as arthritis, arthrosclerosis, and chronic infections [30]. Our findings demonstrate that KYNA significantly and dose-dependently down-regulated LPS-stimulated, but not basal CCL-2 secretion, supporting its role in promoting an anti-inflammatory immune profile. Our results show that KYNA also up-regulates LPS-stimulated M-CSF secretion in THP-1-derived macrophages. M-CSF is a growth factor that regulates the survival, proliferation, and differentiation of monocytes, macrophages, dendritic cells, and osteoclasts [31]. M-CSF-induced macrophages are involved in various pathological conditions, such as cancer, inflammation, and bone disease [32]. Conversely, these cells support immunosuppression, disease resolution, and tissue repair. In several tissue injury models, M-CSF administration enhanced monocyte infiltration, promoting clearance of damaged cells and tissue regeneration [31,32]. As this is the first study demonstrating the effect of KYNA on M-CSF and CCL-2 secretion, the biological significance of this regulation warrants further investigation. KYNA is known to affect the activity of various cells, primarily through activation of the GPR35 and AhR, both of which play key roles in regulating immune responses. GPR35 is predominantly expressed in immune and gastrointestinal tissues, while AhR, a ligand-dependent transcription factor, is broadly distributed across mammalian cells types, including immune cells [9]. To investigate the receptor mechanism by which KYNA influences the production of selected cytokines and chemokines, we used antagonists of GPR35 and AhR. Reports investigating the effects of AhR and GPR35 antagonists on cytokine secretion, or more broadly, on inflammatory processes, remain remarkably scanty. Recently, the GPR35 antagonist ML-145 has been shown to effectively alleviate LPS-induced inflammatory responses in BV-2 microglial cells [33]. Similarly, the AhR antagonist CH-223191 has been reported to reduce a cadmium-induced increase in IL-6 levels in lung leukocytes [34]. On the other hand, CH-223191 was found to compromise the anti-inflammatory effects of indole-3-propionic acid in LPS-treated myocardial cells [35]. Using genetic methods, it was demonstrated that AhR knockout mice are hypersensitive to LPS and that this effect is mitigated by 3-methylcholanthrene, an AhR agonist administered before LPS. Similarly, higher sensitivity to LPS was found in mice with macrophages deficient in AhR. In addition, it was reported that macrophages derived of AhR, knockdown mice responded with increased IL-1beta secretion when exposed to LPS in vitro [36]. Our findings are in perfect agreement with these results indicating the involvement of the AhR receptor in the macrophage response to inflammatory stimulation.

Interestingly, in our study, both antagonists significantly reduced the basal secretion of CCL-2 from the THP-1 macrophages. This result suggests that agonists of both receptors may be either present in the culture medium or produced endogenously during culture at levels sufficient to stimulate CCL-2 secretion. This finding is consistent with the hypothesis of the presence of natural AhR agonists in standard culture media [37]. Importantly, this study also confirmed the functional effectiveness of CH-223191 [37], which is consistent with our results. Furthermore, our results demonstrated that the GPR35 antagonist blocked and partly reduced the KYNA-induced IL-10 and M-CSF secretion, respectively, whereas the AhR antagonist inhibited the effect exerted by KYNA on the IL-6, CCL-2, and M-CSF, but not IL-10 secretion.

Thus, we revealed that in THP-1-derived macrophages kept under inflammatory conditions, the action of KYNA is, at least in part, dependent on the activation of the GPR35 and AhR. Interestingly, KYNA appears to act on both receptors, suppressing the pro-inflammatory cytokine IL-6 while enhancing the anti-inflammatory cytokine IL-10. This dual action may contribute to a pronounced attenuation of inflammation. Therefore, our findings indicate a new therapeutic target(s) in the search for drugs with anti-inflammatory potential. The presented results further suggest the potential therapeutic application of KYNA in attenuating systemic inflammatory responses, a factor of particular relevance in disorders characterized by chronic low-grade inflammation, such as metabolic syndrome, type 2 diabetes, cardiovascular disease, and inflammatory bowel disease. KYNA possesses a favorable pharmacological profile, i.e., it is efficiently absorbed from the gastrointestinal tract, does not cross the blood–brain barrier, is not metabolized, and is rapidly excreted in urine. Moreover, its systemic levels can be easily elevated by injection or oral administration, either as a drug or dietary supplement. This makes KYNA a promising candidate for modulating immune responses in both acute and chronic inflammatory conditions. Indeed, previous studies have proposed dietary KYNA supplementation as a preventive strategy against metabolic syndrome [7], and as a potential adjunctive therapy to slow the progression of colorectal cancer [6]. Whether increasing the circulating KYNA levels, particularly through dietary interventions, could serve as an effective treatment for a broader range of diseases, remains an important and open question that warrants further investigation.

### Limitations

This study provides new insights into the immunomodulatory properties of KYNA in THP-1-derived macrophages and indicates the involvement of AhR and GPR35. However, it has several limitations that warrant consideration

Most notably, the conclusions regarding receptor-mediated mechanisms are based solely on the use of pharmacological antagonists. Although these inhibitors are widely accepted tools in receptor studies, their specificity, potential off-target effects, and incomplete receptor blockade, cannot be excluded. As such, our findings would be significantly strengthened by the inclusion of genetic approaches, such as receptor knockdown or knockout models, to definitively establish the involvement of AhR and GPR35 in mediating KYNA’s effects.

Moreover, the study was conducted exclusively on THP-1-derived macrophages, an immortalized human monocytic cell line. While widely used as a model of human macrophages, THP-1 cells may not fully recapitulate the complexity and heterogeneity of primary human macrophages or tissue-resident macrophage populations. Thus, the results may not be directly translatable to in vivo conditions.

Additionally, all experiments were performed in in vitro conditions, which do not fully reflect the dynamic environment of the immune system in vivo. Factors that may influence macrophage responses, e.g., cell–cell interactions, circulating mediators, and tissue-specific signals, were absent in this system.

## 4. Materials and Methods

### 4.1. Materials

Unless stated otherwise, most of the materials were purchased from Sigma (Darmstadt, Germany).

### 4.2. Cells Handling

Human peripheral blood acute monocytic leukemia cells THP-1 (ATCC, No. TIB-202) were cultured in RPMI 1640 medium supplemented with 10% FBS and antibiotics (100 U/mL penicillin and 100 μg/mL streptomycin. To activate the THP-1 cells into macrophages (THP-derived macrophages), cells growing on a 24-well plate at the density of 5 × 10^5^ cells/well were incubated in the presence of 100 ng/mL of Phorbol 12-myristate 13-acetate (PMA) for 48 h. Differentiated adherent cells were washed with the culture medium and incubated for another 24 h in fresh growing media, without PMA, to obtain resting THP-1-derived macrophages.

### 4.3. KYNA Production

THP-1-derived macrophages were cultured on a 24-well plate at the density of 5 × 10^5^ cells/well. The cells were washed four times with Hank’s balanced salt solution and then incubated with kynurenine (L-KYN) dissolved in Hank’s balanced salt solution at 37 °C in 5% CO_2_.

To assess the effect of substrate concentration on KYNA production, cells were exposed to 2.5, 5, or 10 µM of L-KYN for 2 h. Whereas to evaluate the effect of incubation time, THP-1-derived macrophages were incubated with 5 µM of L-KYN for 1, 2, or 4 h. To examine the influence of 25 µM of AOAA and 10 μg/mL of LPS on KYNA production, cells were incubated with the 5 µM of L-KYN for 2 h. At the end of the incubation time, supernatants were collected, 50% tricarboxylic acid was added to stop the reaction, and precipitated proteins were removed by centrifugation. The supernatants were analyzed for KYNA content, as previously described [38]. Briefly, the supernatants were applied to cation-exchange columns (Dowex 50W^+^) and KYNA content was determined using an HPLC system with fluorescence detection (excitation: 334 nm; emission: 398 nm).

### 4.4. ELISA

The THP-1-derived macrophages growing on a 24-well plate at the density of 5 × 10^5^ cells/well were incubated in the presence of LPS (1 μg/mL), KYNA (50–250 µM), ML-145 (10 μM), and CH-223191 (10 μM) for 24 h. ML-145, an antagonist of GPR35, or CH-223191, an antagonist of the AhR, were added 15 min prior to KYNA and LPS. After the incubation, the culture supernatants were collected, centrifuged, and stored at −80 °C. CCL-2, M-CSF, IL-12, and MMP-9 were measured by means of ELISA kits (R&D Systems, Minneapolis, MN, USA), while IL-6, TNF-α, IL-1β, and IL-10 were measured by ELISA kits (BD Bioscience, Warsaw, Poland), following the manufacturers’ instructions.

In experiments evaluating the effect of KYNA alone, control cells were incubated in culture medium, without KYNA. For experiments assessing the influence of KYNA under LPS stimulation, the control consisted of cells treated with LPS alone. The concentrations of drugs used were chosen based on data from the literature and confirmed in our own preliminary studies.

### 4.5. Data Analysis

The results are presented as the mean ± standard deviation (SD). The significance of differences was determined using GraphPAD Prism 10 (GraphPAD Software Inc., Boston, MA, USA). All the results met the normal distribution criteria therefore the data were analyzed by one-way analysis of variance, followed by the Tukey test; *p* values < 0.05 or less were considered to be significant. Each experimental condition was tested at least in triplicate (*n* = 3) and the experiments independently repeated at least twice (mostly three times).

## 5. Conclusions

In inflammatory conditions, kynurenic acid (KYNA) selectively influenced cytokine and chemokine production. KYNA decreased IL-6 and CCL-2 levels and decreased IL-10 and M-CSF in THP-1-derived macrophages. These effects were receptor-dependent. Specifically, the AhR antagonist CH-223191 attenuated KYNA-induced changes in IL-6 and CCL-2, while the GPR35 antagonist ML-145 blocked the modulation of IL-10. Both receptors contributed to the KYNA-mediated enhancement of M-CSF secretion. Moreover, THP-1-derived macrophages were shown to produce KYNA from L-KYN in a dose- and time-dependent manner. This process was enhanced by LPS and inhibited by AOAA, indicating the involvement of aminotransferase activity. Overall, these results indicate that KYNA exerts differential, receptor-specific immunomodulatory effects on macrophages in inflammatory conditions, supporting its role as a regulator of innate immune responses.

## Data Availability

The data presented in this study are available on request from the corresponding author.

## Supplementary Materials

The following supporting information can be downloaded at: https://www.mdpi.com/article/10.3390/molecules30153133/s1. Table S1. Influence of KYNA on the production of cytokines and chemokines in THP-1-derived macrophages.

## Abbreviations

The following abbreviations are used in this manuscript:

KYNA	kynurenic acid
GPR35	G protein-coupled receptor 35
AhR	aryl hydrocarbon receptor
LPS	lipopolysaccharide
L-KYN	kynurenine
AOAA	aminooxyacetic acid
