# Supplementary material for: The Pharmacological Evidences for the Involvement of AhR and GPR35 Receptors in Kynurenic Acid-Mediated Cytokine and Chemokine Secretion by THP-1-Derived Macrophages
Source: Molecules. 2025 Jul 26;30(15):3133. doi: 10.3390/molecules30153133 (PMC12348619; doi:10.3390/molecules30153133)
Supplement: Supplementary file 1 [file molecules-30-03133-s001.zip › molecules-3762872-supplementary.pdf]

molecules

MDPI

Table S1. Influence of KYNA on the production of cytokines and chemokines in THP-1-derived macrophages. Data are presented in pg/ml; nd - not determined; udl - under detection limit; (*) $p<0.05$; NS - not statistically significant.

		Control	KYNA 50	KYNA 125	KYNA 250
**CCL-2**	mean±SD	38.30±0.98	35.08±1.09	36.85±1.84	37.27±1.42
	p	NS	NS	NS	NS
**IL-1β**	mean±SD	28.15±0.60	27.10±0.05	nd	29.72±0.71
	p	NS	NS		NS
**IL-6**	mean±SD	2.67±0.13	1.75±0.03	1.75±0.70	3.79±0.33
	p	NS	NS	NS	NS
**IL-10**	mean±SD	7.92±1.88	9.79±8.13	13.54±10.63	14.17±5.00
	p	NS	NS	NS	NS
**IL-12**	mean±SD	udl	udl	nd	udl
	p				
**M-CSF**	mean±SD	udl	udl	nd	udl
	p				
**TNF-α**	mean±SD	5.49±0.83	6.13±1.48	4.91±0.26	9.55±0.91
	p	NS	NS	NS	NS

 https://doi.org/10.3390/molecules30153133
